# Root biomass and soil carbon distribution in hybrid poplar riparian buffers, herbaceous riparian buffers and natural riparian woodlots on farmland

**DOI:** 10.1186/2193-1801-2-539

**Published:** 2013-10-17

**Authors:** Julien Fortier, Benoit Truax, Daniel Gagnon, France Lambert

**Affiliations:** Fiducie de recherche sur la forêt des Cantons-de-l’Est / Eastern Townships Forest Research Trust, 1 rue Principale, J0B 2M0 Saint-Benoît-du-Lac, QC Canada; Département des sciences biologiques, Université du Québec à Montréal, C.P. 8888, H3C 3P8 succ. Centre-ville, Montréal, QC Canada; Department of Biology, University of Regina, 3737 Wascana Parkway, S4S 0A2 Regina, SK Canada

**Keywords:** Agroforestry, Afforestation, Agriculture, Land use, Coarse roots, Fine roots, Vertical distribution

## Abstract

The objectives of this study were to compare coarse root (diameter > 2 mm) and fine root (diameter < 2 mm) biomass, as well as distribution of soil carbon stocks in 3 types of riparian land uses across 4 sites located in farmland of southern Québec, Canada: (1) hybrid poplar buffers (9th growing season); (2) herbaceous buffers; (3) natural woodlots (varying in tree species and age). For all land uses most of the root biomass was within the 0–20 cm depth range. Total coarse root biomass, to a 60 cm depth, ranged from 8.8-73.7 t/ha in woodlots, 0.6-1.3 t/ha in herbaceous buffers, and 9.2-27.3 t/ha in poplars. Total fine root biomass ranged from 2.68-8.64 t/ha in woodlots, 2.60-3.29 t/ha in herbaceous buffers, and 1.86-2.62 t/ha in poplars. Total root biomass was similar or higher in poplar buffers compared to a 27 year-old grey birch forest. This indicates that poplar buffers accelerated riparian soil colonisation by roots compared to natural secondary succession. Generally, fine root biomass in the surface soil (0–20 cm) was lower in poplar than in herbaceous buffers; the reverse was observed at greater depth. Highest coarse root biomass in the 40–60 cm depth range was observed in a poplar buffer, highlighting the deep rooted nature of poplars. On average, total soil C stocks (0–60 cm) were greater in woodlots than in riparian buffers. On most sites, soil C stocks tended to be lower in poplar buffers compared to adjacent herbaceous buffers, especially in surface soil, probably because of lower fine root biomass in poplar buffers. Across all sites and land uses, highest soil C stocks at the different soil depths were found in the soil layers of woodlots that also had the greatest fine root biomass. Strong positive linear relationships between fine root biomass and soil C stocks in the 0–20 cm depth range (*R*^2^ = 0.79, p < 0.001), and in the whole soil profile (0–60 cm) (*R*^2^ = 0.65, p < 0.01), highlight the central role of fine root biomass in maintaining or increasing soil C stocks.

## Background

Food security and global sustainability is threatened by the ongoing climate change, which may impact soil erosion processes, crop productivity and soil quality (Lal et al. 2011). In that perspective, management decisions that reduce soil erosion and increase carbon (C) storage to improve soil health will contribute to the resilience of soils and agricultural systems (Lal et al. 2011). In agricultural landscapes, the implementation of agroforestry systems has the potential to provide a very high C sequestration capacity compared to other greenhouse gas mitigation strategies (Jose and Bardhan 2012). Agroforestry systems such as hybrid poplar (*Populus* x spp.) riparian buffers have a high potential to store C in both above and belowground biomass (Tufekcioglu et al. 2003), with biomass C storage ability of poplar buffers being largely affected by site fertility (Fortier et al. 2010b).

While aboveground biomass in poplar plantations and agroforestry systems has been widely studied around the world (Fang et al. 2007; Fortier et al. 2010; Laureysens et al. 2004; Truax et al. 2012; Zabek and Prescott 2006; Christersson 2010), fewer studies have evaluated the belowground biomass of these systems. In 9 year-old cottonwood (*P*. *deltoides*) plantations in India, coarse root biomass decreased with increasing spacing, from 29.8 t ha^-1^ (2 × 2 m spacing) to 5.6 t ha^-1^ (6 × 6 m spacing), while fine root biomass increased with increasing spacing (from 13.8 to 23 t ha^-1^) (Puri et al. 1994). However, Fang et al. (2007) reported little root biomass differences for various spacing in 10 year-old hybrid poplar plantations. Poplar clone selection can also affect belowground biomass growth (Heilman et al. 1994; Al Afas et al. 2008). Stump and large root biomass ranging from 12.3 to 29.6 t ha^-1^ and, small and fine root biomass ranging from 6.6 to 11 t ha^-1^ have been observed for different clones in a closely spaced 4 year-old plantation (10 000 stems ha^-1^) (Heilman et al. 1994). Plantation site quality and management also influence belowground biomass growth. Across 6 mature plantation sites in Sweden (361 to 3279 stems ha^-1^), poplar stump and root biomass respectively ranged from 12.9 to 66.9 t ha^-1^ and 4.7 to 10.9 t ha^-1^ (Johansson and Hjelm 2012). In multi-species riparian buffer combining hybrid poplars and cool-season grass, a total root biomass of 14.3 t ha^-1^ was observed after 7 years (Tufekcioglu et al. 2003). In short, these studies suggest that poplar belowground biomass accumulation can be relatively high, but extremely variable, depending on the poplar system management and design, but also depending on local ecological conditions. Consequently, there is a need to assess belowground biomass in hybrid poplar riparian buffers across different sites in order to refine C stocks estimates in agroforestry systems.

In riparian buffers, root system dimensions and distribution, which vary with plant species (Tufekcioglu et al. 1999), are also known to influence important processes such as nutrient uptake, organic matter supply to soil, soil stabilisation against erosion, channel formation, runoff control, etc. (Dosskey et al. 2010). For example, the use of deep-rooted vegetation is very important for increasing the depth of the active denitrification zone in restored riparian zones, because organic matter supply at depth is highly dependent upon soil colonisation by roots (Gift et al. 2008). Deep rooted tress will also uptake nutrients and water at greater soil depth than herbaceous vegetation (Schultz et al. 1995). While most studies have concluded that the majority of poplar coarse and fine roots in plantations and agroforestry systems is located near the soil surface (Douglas et al. 2010; Tufekcioglu et al. 1999; Puri et al. 1994; Al Afas et al. 2008), poplar roots can extend to more than 3 m deep into soil after only 4 years (Heilman et al. 1994).

Although soil colonisation by roots may affect important riparian buffer functions, very few studies have evaluated root distribution in mature hybrid poplar riparian buffers across different agricultural sites. It is also important to compare belowground biomass distribution of riparian agroforestry systems with the riparian land use they replaced (herbaceous buffers, row crops, hayfields, pastures) (Tufekcioglu et al. 1999). Locally, poplar plantation attributes can also be compared to those of woodlots in order to evaluate how different or similar are planted poplars stands from adjacent naturally regenerated stands (Boothroyd-Roberts et al. 2013; Coleman et al. 2004).

Unlike C storage in root biomass, soil C storage capacity of agroforestry and afforested systems is less clear. In the case of poplar afforestation and agroforestry, C sequestration in terms of soil C increases remains uncertain, with studies reporting contrasting results, sometimes showing positive, negative, or no impacts (Arevalo et al. 2009; Mao et al. 2010; Coleman et al. 2004; Boothroyd-Roberts et al. 2013; Peichl et al. 2006; Sartori et al. 2007; Teklay and Chang 2008). This is possibly because of the high impact of land use changes (from abandoned field, pasture, row crop, or grassland to plantation), plantation management (rotation length, site preparation, tending operations, fertilisation, etc.) and local conditions on soil C stocks and dynamics (Coleman et al. 2004; Laganière et al. 2010; Sartori et al. 2007; Teklay and Chang 2008; Guo and Gifford 2002). It was also suggested that short rotation poplar plantations generally contained less soil C, especially at depth, when compared to adjacent woodlots (Coleman et al. 2004). Globally, soils store a larger quantity of C than plant biomass and the atmosphere combined (Jobbagy and Jackson 2000), and a land use change from agriculture to agroforestry or afforestation can have important impacts on soil C stocks and dynamics (Guo and Gifford 2002). In that perspective, the potential of poplar riparian buffers to store soil C in replacement of widespread herbaceous buffers, needs to be evaluated.

The objectives of this study were to compare coarse root (diameter > 2 mm) and fine root (diameter < 2 mm) biomass, as well as distribution of soil carbon stocks in 3 types of riparian land uses across 4 sites located in farmland of southern Québec, Canada: (1) hybrid poplar riparian buffers (9^th^ growing season); (2) herbaceous riparian buffers; (3) natural riparian woodlots (varying in tree species and age).

## Results

### Riparian land use soil characteristics

Results in Table 1 suggest large variation in soil pH and bulk density (BD) among riparian land use types at each site. Across the 4 sites, woodlot surface soils tend to be more acid and less compact (in terms of BD) than riparian buffer soils. Soil pH in the 0–20 cm depth interval ranged from 4.25 to 5.35 in woodlots, from 5.44 to 6.37 in poplar buffers and from 5.48 to 7.23 in herbaceous buffers, while bulk density (0–20 cm) ranged from 0.66 to 1.16 in woodlots, from 0.90 to 1.23 in poplar buffers and from 0.90 to 1.22 in herbaceous buffers (Table 1).

**Table 1:** **Soil profile characteristics of three riparian land uses at four sites**

		pH			Bulk density (g cm^-3^)			Stoniness (%)			Texture (%)(clay-silt-sand)		
Land uses	Sites	0-20 cm	20-40 cm	40-60 cm	0-20 cm	20-40 cm	40-60 cm	0-20 cm	20-40 cm	40-60 cm	0-20 cm	20-40 cm	40-60 cm
Woodlot - Hemlock	Brompton	5.35	6.50	6.80	0.76	1.21	1.54	0	0	0	17-44-39	27-27-46	21-18-61
Woodlot - White cedar	Magog	4.53	4.95	5.45	0.85	0.86	0.92	15	12	14	25-34-41	13-30-57	13-28-60
Woodlot - Grey birch	Roxton	4.93	5.15	5.18	1.16	1.33	1.32	0	0	0	16-37-47	18-43-39	21-39-40
Woodlot - Sugar maple	St-Isidore	4.25	4.80	4.90	0.66	0.81	0.99	1	1	10	15-42-43	11-52-37	13-50-37
Hybrid poplar buffer	Brompton	6.37	6.18	6.22	1.14	1.33	1.22	0	0	0	15-38-47	11-44-45	11-33-56
Hybrid poplar buffer	Magog	5.63	5.75	5.85	0.90	1.00	0.87	12	19	35	16-35-49	16-33-51	14-32-54
Hybrid poplar buffer	Roxton	6.18	6.09	6.20	1.23	1.35	1.38	1	3	4	19-29-52	17-35-48	28-26-46
Hybrid poplar buffer	St-Isidore	5.44	5.78	5.95	1.07	1.03	0.92	2	15	36	22-40-38	13-46-40	16-39-45
Herbaceous buffer	Brompton	6.15	5.73	5.95	0.90	1.21	1.32	0	0	0	16-36-48	13-58-29	17-56-27
Herbaceous buffer	Magog	5.73	6.10	6.18	1.12	1.08	1.10	13	3	0	13-32-55	8-15-77	11-10-79
Herbaceous buffer	Roxton	7.23	6.30	6.35	1.22	1.35	1.40	0	0	0	16-31-53	13-24-63	12-11-77
Herbaceous buffer	St-Isidore	5.48	5.78	6.03	0.92	0.98	0.94	1	38	40	21-50-29	13-56-31	13-32-55
Land use × Site	*p*<	0.01	0.001	0.001	0.01	NS	NS	NS	0.001	0.05			
	SE	0.16	0.17	0.13	0.06	0.09	0.09	3	4	7			
Land use	*p*<	0.001	0.001	0.001	0.001	NS	NS	NS	0.05	0.05			
	SE	0.08	0.09	0.06	0.03	0.04	0.05	2	2	3			
Site	*p*<	0.001	0.001	0.001	0.001	0.001	0.001	0.001	0.001	0.001			
	SE	0.09	0.10	0.07	0.03	0.05	0.05	2	2	4			

In addition, soil pH tends to substantially increase with depth in woodlots (especially in older stands), while soil pH shows little or no decrease with depth across the four sites in both types of riparian buffers (Table 1). A similar trend is observed for bulk density. In other words, soil properties (pH and BD) in poplar and herbaceous buffers tend to be much more homogeneous down the soil profile, when compared to older riparian woodlot soils (hemlock, cedar and sugar maple) (Table 1). Relatively large volumes of stones (up to 40%) where found, especially at greater depth, in the poplar and the herbaceous buffers of the St-Isidore site and in the poplar buffer at Magog (Table 1).

### Root biomass distribution

**Figure 1:**
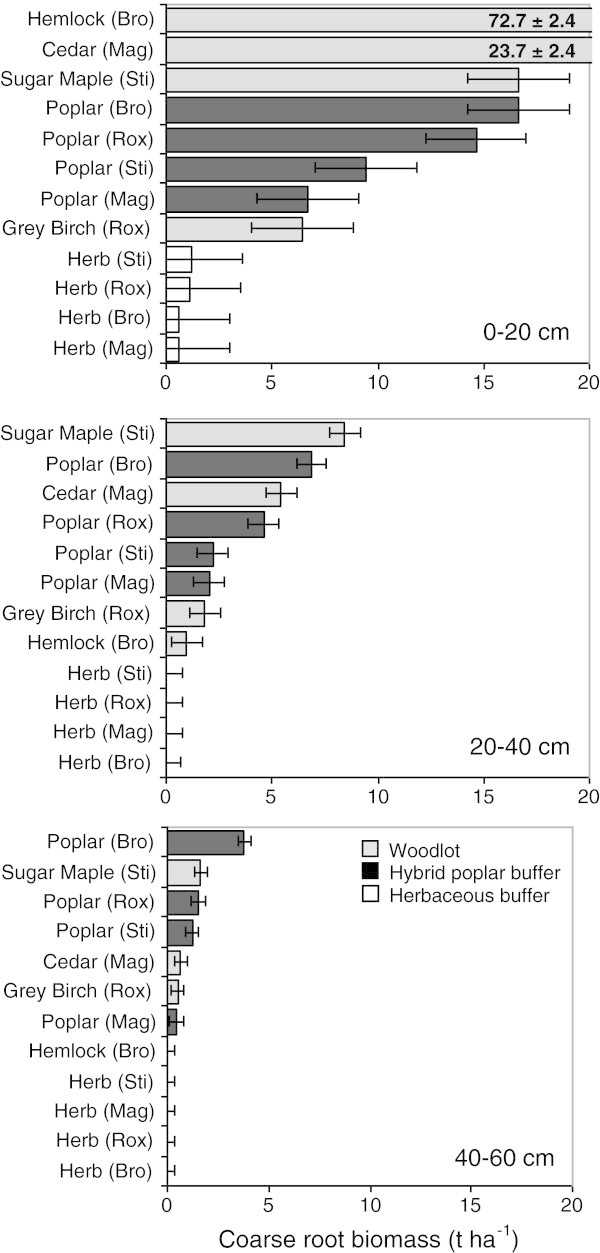
**Coarse root biomass vertical distribution (t ha** ^**-1**^ **) for three soil depths (0–20, 20–40 and 40–60 cm) for three different riparian land uses at four sites.** Site × Land use interaction is significant at p < 0.001 for the three soil depths. Horizontal bars represent SE.

**Figure 2:**
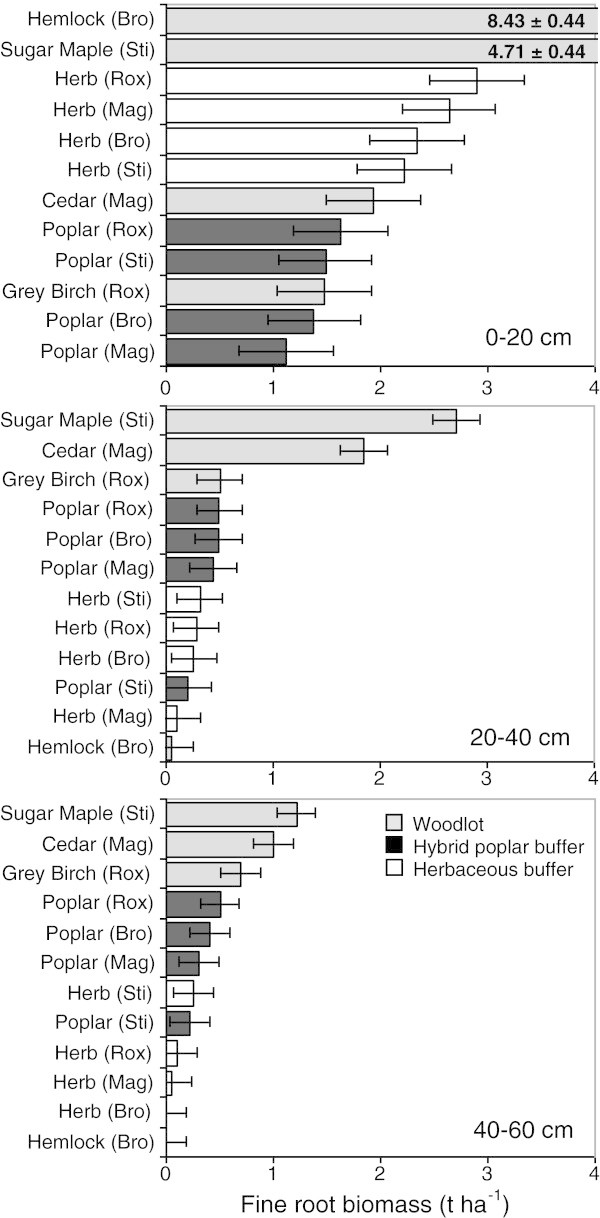
**Fine root biomass vertical distribution (t ha** ^**-1**^ **) for three soil depths (0–20, 20–40 and 40–60 cm) for three different riparian land uses at four sites.** Site × Land use interaction is significant at p < 0.001 for the 0–20 cm and 20–40 cm soil depths, and at p < 0.05 for the 40–60 soil depth. Horizontal bars represent SE.

A significant Land use × Site interaction was observed for coarse root biomass (> 2 mm) and fine root biomass (< 2 mm) at each depth range and in the whole soil profile (Figures 1 and 2, Table 2). Total coarse root biomass ranged from 8.8-73.7 t ha^-1^ in woodlots, 0.6-1.3 t ha^-1^ in herbaceous buffers, and 9.2-27.3 t ha^-1^ in poplar buffers (Table 2). Total fine root biomass ranged from 2.68-8.64 t ha^-1^ in woodlots, 2.60-3.29 t ha^-1^ in herbaceous buffers, and 1.86-2.62 t ha^-1^ in poplar buffers (Table 2). Across all land uses and sites, most coarse and fine root biomass was located near the soil surface (0–20 cm depth range) (Figures 1 and 2, Table 2). Percentage of coarse roots located in 0-20 cm soil depth interval ranged from 62-99% in woodlots, 94-100% in herbaceous buffers, and 61 to 73% in poplar buffers (Table 2). The greatest decrease in coarse and fine root biomass down the soil profile was observed in the oldest woodlot (hemlock) and in the herbaceous buffers (Table 2). The highest coarse root biomass in the deepest soil depth range studied (40–60 cm) was observed in the poplar buffer at the Bromptonville site (Figure 1). Compared to the early successional stand (27 year-old grey birch woodlot), total root biomass was similar or higher in poplar buffers (Table 2).

**Table 2:** **Coarse and fine root biomass (t ha** ^**-1**^ **) to 60 cm soil depth for three land uses at four sites**

		Coarse root biomass				Fine root biomass				Total root biomass
Land uses	Sites	Total (t ha^-1^)	0-20 cm (%)	20-40 cm (%)	40-60 cm (%)	Total (t ha^-1^)	0-20 cm (%)	20-40 cm (%)	40-60 cm (%)	(t ha^-1^)
Woodlot - Hemlock	Brompton	73.7	99	1	0	8.48	99	1	0	82.2
Woodlot - White cedar	Magog	29.8	80	18	2	4.77	40	39	21	34.6
Woodlot - Grey birch	Roxton	8.8	73	21	6	2.68	55	19	26	11.4
Woodlot - Sugar maple	St-Isidore	26.7	62	32	6	8.64	55	31	14	35.3
Hybrid poplar buffer	Brompton	27.3	61	25	14	2.28	61	22	18	29.6
Hybrid poplar buffer	Magog	9.2	73	22	5	1.86	60	24	16	11.0
Hybrid poplar buffer	Roxton	20.8	70	22	7	2.62	62	19	19	23.4
Hybrid poplar buffer	St-Isidore	12.9	73	17	10	1.91	78	11	11	14.8
Herbaceous buffer	Brompton	0.6	100	0	0	2.60	90	10	0	3.2
Herbaceous buffer	Magog	0.6	99	1	0	2.80	94	4	2	3.4
Herbaceous buffer	Roxton	1.1	98	2	0	3.29	88	9	3	4.4
Herbaceous buffer	St-Isidore	1.3	94	3	2	2.79	80	11	9	4.0
SE		2.5				0.45				2.6
*p*<		0.001				0.001				0.001

Vertical distribution of coarse and fine root biomass in 20 cm intervals expressed as percentages of total root biomass.

**Figure 3:**
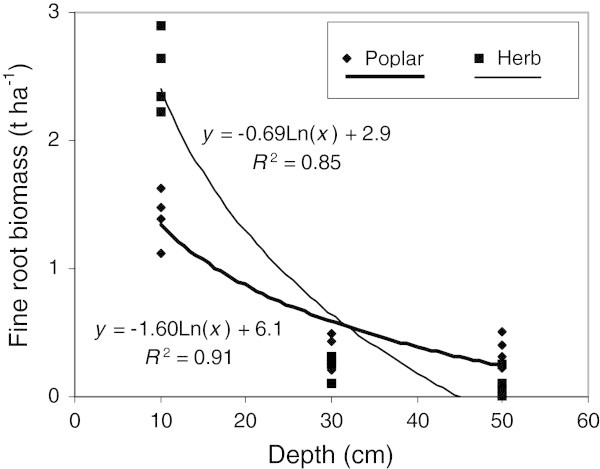
**Logarithmic relationships between soil depth (cm) and fine root biomass (t ha** ^**-1**^ **) for hybrid poplar (Poplar) and herbaceous (Herb) riparian buffers.** Both relationships are significant at *p* < 0.001. Mean fine root biomass for both land uses at each site and at each depth were used as response variables and mid-points of depth intervals were used as predictor variables. For each relationship n = 12.

Results in Figure 1 suggest that coarse root biomass is much greater in poplar buffers lower down the soil profile compared to herbaceous buffers. However, fine root biomass in surface soil (0–20 cm) tends to be lower in poplar buffers than in herbaceous buffers, while the reverse is observed at greater depth (Figure 2). These observations are supported by significant relationships between soil depth and fine root biomass (Figure 3). These relationships suggest that below 30 cm of depth (Figure 3), fine root biomass becomes greater in poplar buffers than in herbaceous buffers, while the reverse is observed above this depth. Still, for the different depth intervals, fine root biomass was always the greatest in one or several woodlots compared to both types of riparian buffers (Figure 2).

### Soil carbon stocks distribution

**Figure 4:**
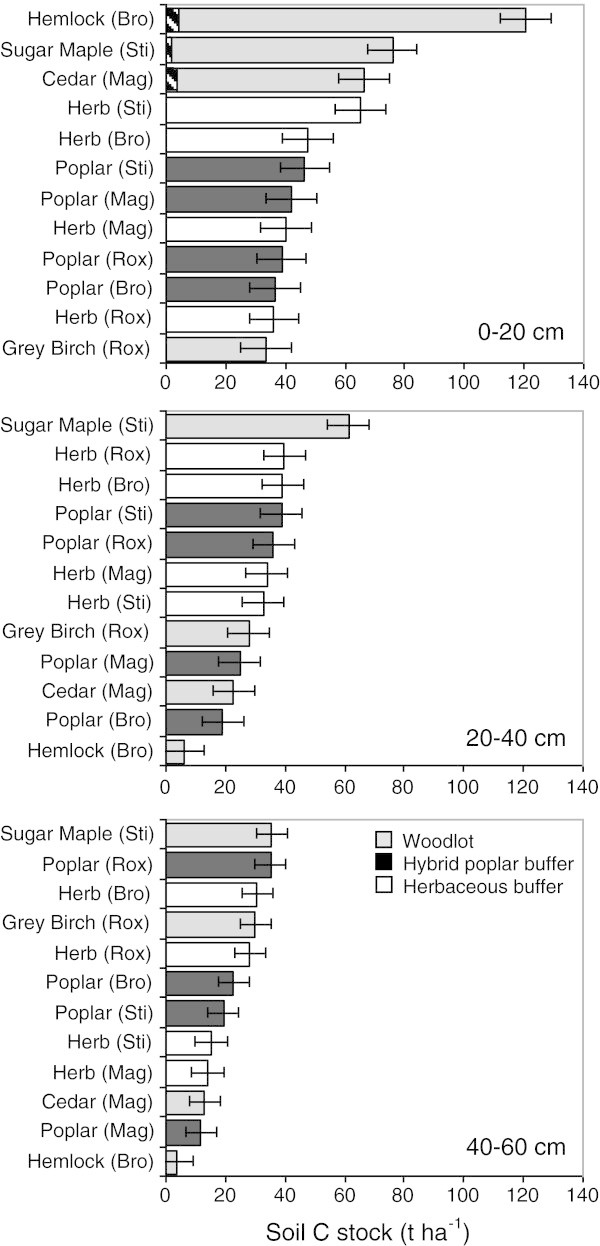
**Soil C stock vertical distribution (t ha** ^**-1**^ **) for three soil depths (0–20, 20–40 and 40–60 cm) for three different riparian land uses at four sites.** Site × Land use interaction is significant at p < 0.001 for the 0–20 cm soil depth, and at p < 0.01 for the 20–40 and 40–60 cm soil depths. Stripped shading in top diagram represents C in the LFH (O horizon). Horizontal bars represent SE.

Soil C concentrations near the soil surface (0–20 cm) tend to be highly variable across the different land uses and sites. Soil C concentrations ranged from 14.8-75.5 g kg^-1^ in woodlots, 14.6-35.9 g kg^-1^ in herbaceous buffers, and 16.1-26.0 g kg^-1^ in poplar buffers (Table 3). This large variation was also observed for surface soil C stocks (0–20 cm) (Figure 4). The greatest C concentration and stocks in surface soil (0–20 cm) were found in the older woodlots (hemlock, sugar maple and cedar) and in the herbaceous buffer of St-Isidore-de-Clifton (Figure 4, Table 3). A very large decrease in soil C concentration and stocks with depth was observed in the hemlock woodlot, which has 89% of its soil C stocks located between 0 and 20 cm of depth (Figure 4, Tables 3 and 4). For the other woodlots and for the herbaceous and poplar buffers the decrease in soil C with depth was less abrupt, and sometimes very minor as observed in both buffer types at Roxton Falls (Figure 4, Table 4).

**Table 3:** **Vertical distribution of soil carbon concentration (g kg** ^**-1**^ **) in three riparian land uses at four sites**

		Soil C (g kg^-1^)		
Land uses	Sites	0-20 cm	20-40 cm	40-60 cm
Woodlot - Hemlock	Brompton	75.5	2.5	1.3
Woodlot - White cedar	Magog	43.8	16.5	8.2
Woodlot - Grey birch	Roxton	14.8	11.5	11.9
Woodlot - Sugar maple	St-Isidore	57.8	38.3	19.1
Hybrid poplar buffer	Brompton	17.7	7.3	9.5
Hybrid poplar buffer	Magog	26.0	15.7	11.4
Hybrid poplar buffer	Roxton	16.1	13.9	13.2
Hybrid poplar buffer	St-Isidore	22.8	22.7	16.9
Herbaceous buffer	Brompton	26.5	19.8	13.3
Herbaceous buffer	Magog	20.0	16.0	6.3
Herbaceous buffer	Roxton	14.6	14.8	10.4
Herbaceous buffer	St-Isidore	35.9	27.0	15.6
SE		5.0	4.0	-
*P*		0.001	0.05	NS

**Table 4:** **Total soil carbon (t ha** ^**-1**^ **) to 60 cm soil depth for three land uses at four sites**

Land uses	Sites	Total soil C (t ha^-1^)	LFH (%)	0-20 cm (%)	20-40 cm (%)	40-60 cm (%)
Woodlot - Hemlock	Brompton	130	3	89	5	3
Woodlot - White cedar	Magog	102	4	61	22	13
Woodlot - Grey birch	Roxton	91	0	37	30	33
Woodlot - Sugar maple	St-Isidore	173	1	43	35	21
Hybrid poplar buffer	Brompton	78	0	47	24	29
Hybrid poplar buffer	Magog	78	0	53	32	15
Hybrid poplar buffer	Roxton	110	0	35	33	32
Hybrid poplar buffer	St-Isidore	104	0	45	37	18
Herbaceous buffer	Brompton	117	0	41	33	26
Herbaceous buffer	Magog	88	0	46	38	16
Herbaceous buffer	Roxton	104	0	35	38	27
Herbaceous buffer	St-Isidore	113	0	58	29	13
SE		13				
p<		0.05				

Vertical distribution of soil carbon in 20 cm intervals expressed as percentages of total soil carbon.

Overall, total C stocks (0–60 cm) ranged from 91–173 t ha^-1^ in woodlots, 88–117 t ha^-1^ in herbaceous buffers, and 78–110 t ha^-1^ in poplar buffers. At some sites, C stocks and concentration were significantly higher in the surface and intermediate soil depths of herbaceous buffers than in those of poplar buffers. This was the case at St-Isidore-de-Clifton for the 0–20 cm depth interval and at Bromptonville for the 20–40 cm depth interval (Figure 4). For the whole profile (0–60 cm), soil C stocks were also greater in the herbaceous buffer at Bromptonville when compared to the poplar buffer.

**Figure 5:**
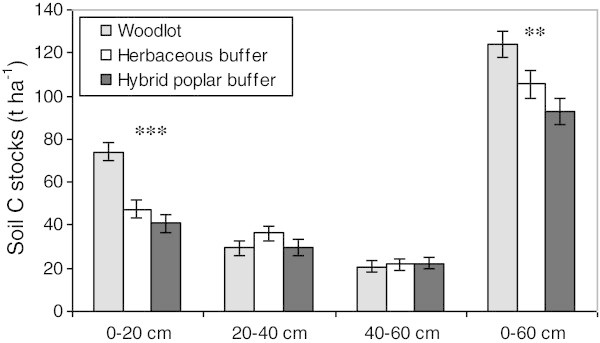
Land use effect on mean soil C stocks for the different soil depth intervals (*** p < 0.001, ** p < 0.01). Vertical bars represent SE.

**Figure 6:**
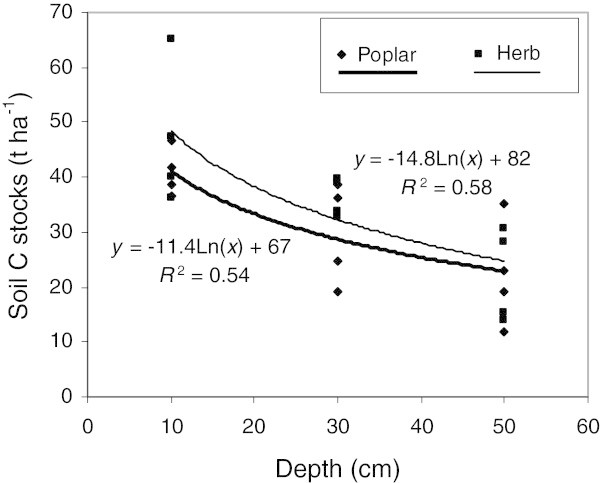
**Logarithmic relationships between soil depth (cm) and soil C stocks (t ha** ^**-1**^ **) for hybrid poplar (Poplar) and herbaceous (Herb) riparian buffers.** Both relationships are significant at *p* < 0.01. Mean C stocks for both land uses at each site and at each depth were used as response variables and mid-points of depth intervals were used as predictor variables. For each relationship n = 12.

At the land use level, significant Land use effects were observed with woodlots having greater soil C stocks, on average, for the 0–20 cm and the 0–60 cm depth intervals compared to both types of buffers (Figure 5). On average, for the whole soil profile (0–60 cm), herbaceous buffers tend to have greater soil C stocks, although they are not statistically different from C stocks of hybrid poplar buffers. Significant relationships between soil depth and C stocks also suggest that herbaceous buffers tend to have more soil C than hybrid poplar buffers, especially near the soil surface (Figure 6).

### Relationship between soil C, fine root biomass and pH

**Figure 7:**
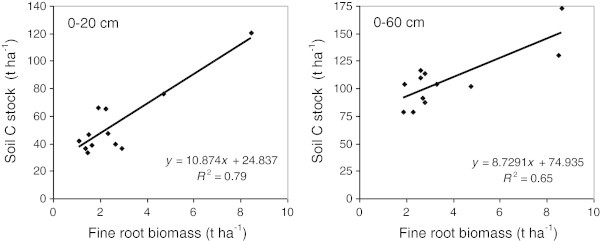
**Linear relationship between fine root biomass (t ha** ^**-1**^ **) and soil C stocks (t ha** ^**-1**^ **) measured in the 0–20 cm soil depth range (left diagram; p < 0.001) and in the entire 0–60 cm soil profile (right diagram; p < 0.01).** Mean fine root biomass data and mean C stocks data obtained for each land use at each site (n = 12) were used to obtain these relationships.

**Figure 8:**
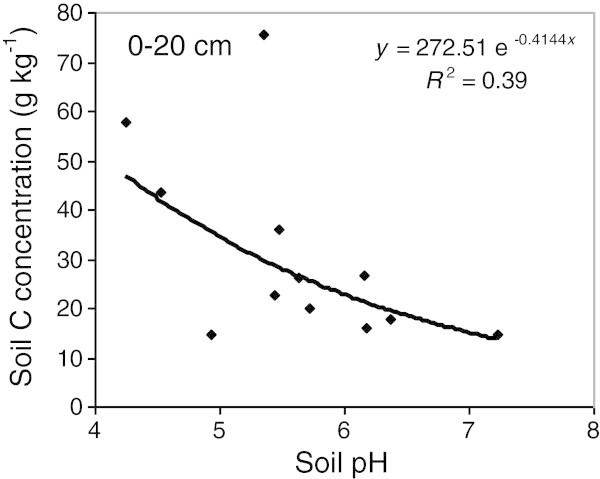
**Negative relationship between soil pH and soil C concentration (excluding LFH) in the 0–20 cm depth range (p < 0.05).** Mean soil pH data and mean C concentration data obtained for each land use at each site (n = 12) were used to obtain this relationship.

Across all sites and land uses, a strong positive linear relationship was observed between fine root biomass and soil C stocks in the 0–20 cm depth interval (*R*^2^ = 0.79, p < 0.001) (Figure 7). A strong positive linear relationship was also observed between those two variables measured in the whole soil profile (0–60 cm) (*R*^2^ = 0.65, p < 0.01) (Figure 7). Finally, a negative relationship between soil pH and soil C concentration in the 0–20 cm depth interval was observed (*R*^2^ = 0.39, p < 0.05) (Figure 8).

## Discussion

### Root biomass in the different riparian land uses

This study suggests that the greatest benefits of establishing an agroforestry system such as a hybrid poplar buffer, in a riparian zone previously dominated by herbaceous vegetation, are (1) an important increase in coarse root biomass down in the soil profile (Figures 1 and 3, Table 2) and (2) an increase in fine root biomass at greater depth in the soil profile (40–60 cm) (Figures 2 and 3). During the 9th growing season, total root biomass in poplar buffers also varied greatly across sites, ranging from 11.0 to 29.6 t ha^-1^, with the greatest biomass observed at the fertile site of Bromptonville, and the lowest at the low fertility site of Magog (Table 2) (Fortier et al. 2013). Although most poplar root system biomass was located near the soil surface (0–20 cm) (Figures 1 and 2, Table 2), results also highlight the particular ability of poplar to colonise deeper soil horizons. At Bromptonville, where hybrid poplar grew best after 9 years (Table 5) (Fortier et al. 2013), a coarse root biomass of 3.8 t ha^-1^ was observed in the 40–60 depth interval; a coarse root biomass that was greater than in any woodlot studied (Figure 1). Results from Heilman et al. (1994) also highlight the deep rooted nature of poplars with significant root biomass being observed below 3 m depth after 4 growing seasons under high intraspecific competition. Hybrid poplars have a root distribution that is typical of early successional species such as aspen (*P*. *tremuloides*), which are generally more deeply rooted than mid successional and climax species (Gale and Grigal 1987). By establishing a deep rooting system, *Populus* species can effectively exploit an unoccupied and more homogeneous soil substrate, which is generally observed following a disturbance (Gale and Grigal 1987). In this study, riparian soil properties (pH, bulk density, and soil C) were more homogeneously distributed with depth in buffer soils than in older woodlot soils (Tables 1, 3 and 4, Figure 4), probably because of recent agricultural activities. The establishment of poplar buffers on those homogeneous riparian soils seems ecologically sound in order to increase the rooting depth of a buffer and subsurface nutrient interception. In comparison, naturally established herbaceous buffers generally have little root biomass at greater depth (40–60 cm) (Figures 1, 2 and 3, Table 2) and therefore occupied a much smaller soil volume.

**Table 5:** **Characteristics of three types of land use at four sites and DBH range of trees sampled for root biomass**

Land uses	Sites	Description	Forest vegetation zone	Elev. (m)	Age of dominant trees (yrs)	DBH range (cm) of sampled trees
Woodlot – Hemlock	Brompton	Primary forest	Sugar maple-Basswood	200	200	20.3-27.6
Woodlot - White cedar	Magog	Secondary forest - livestock access	Sugar maple-Basswood	220	72	24.6-33.3
Woodlot - Grey birch	Roxton	Secondary forest	Sugar maple-Basswood	145	27	6.9-12.9
Woodlot - Sugar maple	St-Isidore	Secondary forest	Sugar maple-Yellow birch	420	54	11.6-16.8
Hybrid poplar buffer	Brompton	Riparian buffer in pasture	Sugar maple-Basswood	140	9	17.5-32.3
Hybrid poplar buffer	Magog	Riparian buffer in pasture	Sugar maple-Basswood	210	9	9.8-20.3
Hybrid poplar buffer	Roxton	Riparian buffer in hayfield	Sugar maple-Basswood	145	9	13.4-22.9
Hybrid poplar buffer	St-Isidore	Riparian buffer in pasture	Sugar maple-Yellow birch	360	9	14.8-24.3
Herbaceous buffer	Brompton	Riparian buffer in pasture	Sugar maple-Basswood	140	-	-
Herbaceous buffer	Magog	Riparian buffer in pasture	Sugar maple-Basswood	205	-	-
Herbaceous buffer	Roxton	Riparian buffer in annual crop	Sugar maple-Basswood	145	-	-
Herbaceous buffer	St-Isidore	Riparian buffer in pasture	Sugar maple-Yellow birch	380	-	-

**Figure 9:**
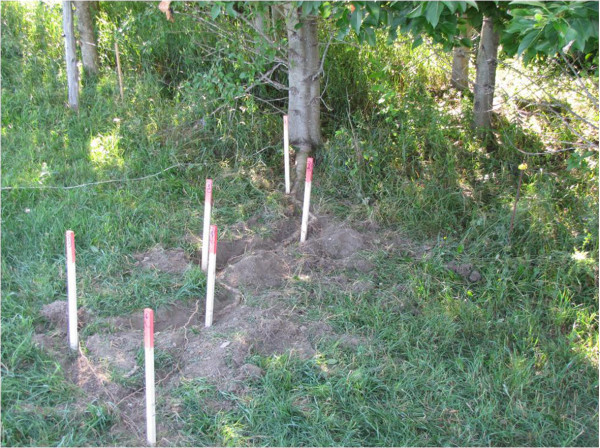
**Lateral roots of hybrid poplar expanding several meters away from the riparian buffer zone into the adjacent pasture.**

The deep rooting system of hybrid poplars may be important for increasing the depth of the active denitrification zone, because organic matter supply at greater soil depth is highly dependent on the colonisation of the soil by roots (Gift et al. 2008). Roots of hybrid poplars and other tree species can also expand several meters away from the trunk and uptake nutrients directly in the adjacent pasture or crop field (Figure 9) (Addy et al. 1999). These lateral roots substantially widen the zone of influence of tree riparian buffers. Moreover, along unstable agricultural streams, deep rooted trees such as poplars may be more efficient than herbaceous vegetation at reducing stream bank erosion (Zaimes et al. 2004). It is also important to highlight that total root biomass was similar or higher in 9 year-old poplar buffers (11.0-29.6 t ha^-1^) compared to the 27 year-old woodlot dominated by grey birch (11.4 t ha^-1^) (Table 2), a typical coloniser of moist areas of abandoned fields in southern Québec (Farrar 2006). At the Roxton Falls site, the 9 year-old hybrid poplar buffer had double the total root biomass found in the grey birch woodlot. Therefore, hybrid poplar buffers accelerated riparian soil colonization by roots compared to natural secondary succession over more than 27 years after agriculture abandonment.

**Figure 10:**
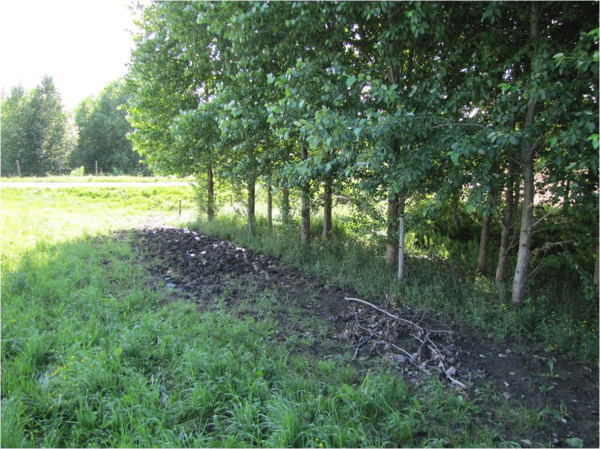
**During the 9th growing season, hybrid poplar roots blocked a sub-surface drain, creating a wet zone at the pasture / riparian buffer interface.**

However, hybrid poplars can also cause disservices to farmers that have sub-surface soil drainage systems. At the St-Isidore-de-Clifton site, it took less than 9 years to have such a drain blocked by poplar roots (Figure 10). While drain blockage may reduce crop yield, it may also recreate a wet zone where other ecosystem services may be supported (denitrification or habitat for wetland species). Tile drains have been long known to substantially reduce riparian buffer effectiveness for non-point source pollution control, as they often bypass the buffer zone (Osborne and Kovacic 1993). Hence, drain blockage by poplar roots could increase buffer performances by reducing hydrological connectivity between the drainage system and surface water.

This study also shows that root biomass distribution in poplar buffers sharply contrasts with the distribution pattern observed in the oldest woodlot, where the hemlock root system is almost entirely restricted to the soil surface (0–20 cm) (Figures 1 and 2, Table 2). This is because in late successional or climax stands, soil nutrients and carbon, mainly originating from root detritus and litter, are concentrated near the soil surface (Gale and Grigal 1987) (Figure 4, Table 4). Consequently, shallow rooted trees such as hemlock are well adapted to such site conditions, were nutrient cycling occurs in a closed loop with little nutrient leakage (Odum 1969). However, more nutrient demanding sub-climax species such as sugar maple, may have a vertical root distribution that is similar to that of pioneer species (Gale and Grigal 1987), as also observed in this study (Figures 1 and 2, Table 2).

**Figure 11:**
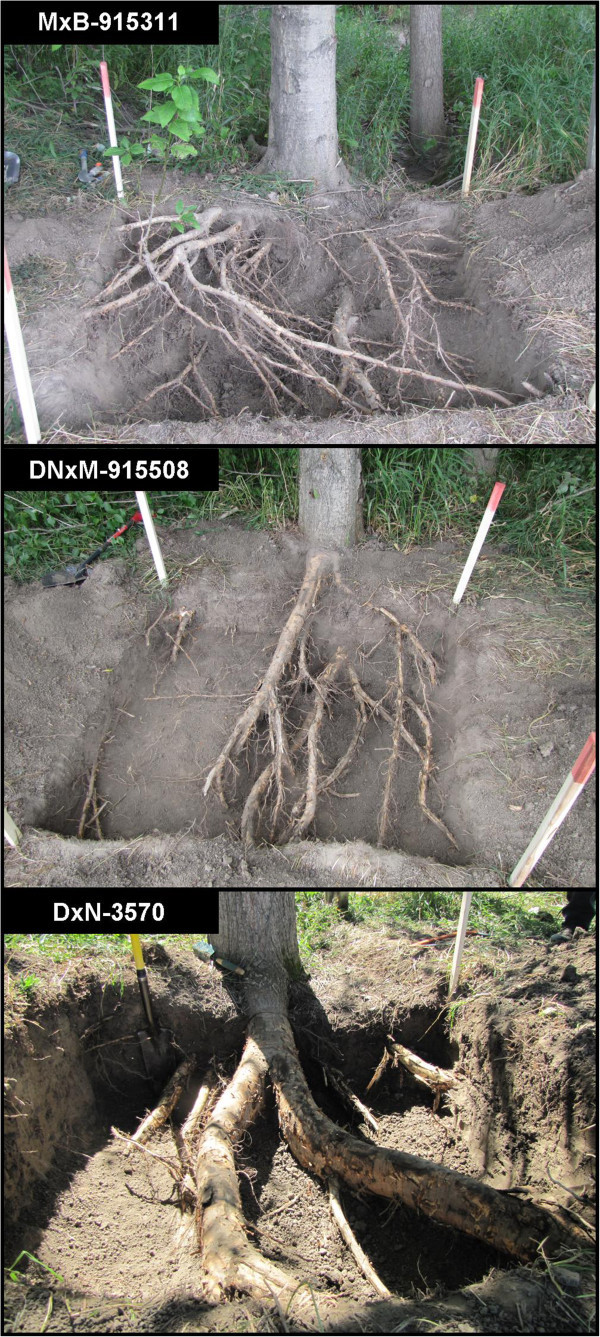
**Excavations exposing coarse roots of three hybrid poplar clones (9th growing season), at the interface of a crop field and a riparian buffer zone.**

Finally, to qualitatively evaluate differences in coarse root architecture of hybrid poplars, we undertook larger excavations (1.5 m × 1.5 m × 0.6 m of depth) on a single tree of the three different clones (Figure 11). Coarse root structure appears to vary greatly with genotype, with clone DxN-3570 having fewer much larger horizontal coarse roots, whereas clones MxB-915311 and DNxM-915508 have a far more ramified smaller coarse root system near the tree base.

### Soil carbon in the different riparian land uses

In general, soil C stocks and concentrations were similar or lower in poplar buffers when compared to adjacent herbaceous buffers (Figure 4, Tables 3 and 4). For the whole soil profile studied (0–60 cm), site comparisons suggest that poplar buffers caused insignificant C gains of 4 t ha^-1^ at Roxton Falls, insignificant C losses of 9 and 10 t ha^-1^ at St-Isidore-de-Clifton and Magog, but significant losses of 39 t ha^-1^ at Bromtonpville (Table 4). Site comparisons also suggest lower C stocks in poplar buffers for the 0–20 cm depth range at St-Isidore-de-Clifton and for the 20–40 cm depth range at Bromptonville (Figure 4). Finally, the regression analysis between soil depth and soil C stocks suggests that the establishment of a hybrid poplar buffer in a riparian zone previously dominated by perennial herbaceous vegetation may result in a decrease in soil C, but mostly near the soil surface (Figure 6). This evidence is consistent with previously published studies. In meta-analyses, it was reported that soil C stocks generally decrease or are unaffected when tree plantations are established in pastures or grassland (Guo and Gifford 2002; Laganière et al. 2010). Surface soil C was also similar when 10 year-old hybrid poplar plantations of southern Québec were compared to adjacent abandoned fields (Boothroyd-Roberts et al. 2013). In Alberta (Canada), no significant differences in soil C stocks (0–50 cm) were reported when 2 and 9 year-old poplar plantations were compared to adjacent land uses (agriculture, grassland, and native aspen) (Arevalo et al. 2009). In chronosequences, a decadal time scale was insufficient to measure significant changes in soil C of poplar plantations (Sartori et al. 2007; Teklay and Chang 2008). Across 27 sites of the North Central United States, paired comparisons found few soil C differences between poplar plantations and agricultural crops (Coleman et al. 2004). On marginal agricultural land in China, a decrease in soil C was observed after 10 years of poplar culture, but an increase in soil C was reported after 20 years, with a recovery time of about 15 years (Mao et al. 2010).

Briefly, this evidence suggests that managing poplar plantations and agroforestry systems on longer rotations (more than 15 years), will probably be needed for soil C sequestration to occur, as also observed for fast-growing *Eucalyptus* plantations established on pastures (Berthrong et al. 2012). In addition, measuring soil C over the first rotation might also yield different results since substantial root biomass will be decomposing and contributing to soil C pools at different soil depths after harvest (Table 2) (Zan et al. 2001). It has also been suggested that intensive pre-planting disturbances such as intensive mechanical site preparation could result in a soil C loss in young plantations (Laganière et al. 2010; Shi et al. 2010). However, this explanation does not hold for interpreting our results because no mechanical site preparation was done, and only a single local herbicide application (1 m^2^/tree) was done to control weeds in the first year (Fortier et al. 2010a).

The trend towards lower or similar soil C stocks found in poplar buffers versus adjacent herbaceous buffers, especially near the soil surface (Figures 4 and 5), might be related to the lower fine root biomass in the surface soil (0–20 cm) of poplar buffers (Figures 2 and 3). Fine root biomass in the surface soil probably decreased as a result of hybrid poplars shading the herbaceous vegetation, because canopy closure in these poplar buffers resulted in a large decrease in understory vegetation biomass after 6 years (Fortier et al. 2011). In addition, compared to trees, herbaceous vegetation is known to allocate a much larger proportion of assimilated C to the root system (Kuzyakov and Domanski 2000), while having higher root turnover (Guo et al. 2007). On the other hand, the dense root mat observed in herbaceous communities near the soil surface may reduce gas and water exchanges (Yakimenko 1998), which may slow down organic matter decomposition in herbaceous buffers. Strong positive linear relationships were observed (across all land uses and sites) between fine root biomass and soil C stocks in the 0–20 cm depth range (*R*^2^ = 0.79, p < 0.001) and in the whole soil profile (0–60 cm) (*R*^2^ = 0.65, p < 0.01) (Figure 7). These relationships highlight the central role of fine root biomass in maintaining or increasing soil C stocks, as previously observed in forest ecosystems (Persson 2012). Highest soil C stocks in the different soil depths sampled were also found in the soil depth of woodlots that had the highest fine root biomass (Figures 2 and 4).

The greatest negative impact of poplar plantations on soil C have been observed on most fertile sites (Coleman et al. 2004), as observed at the very fertile site of Bromptonville (Table 4), where poplar yield was the highest (Fortier et al. 2010; Fortier et al. 2013). It was also at the Bromptonville site that the lowest understory biomass (mainly herbaceous species) was observed during the 6^th^ growing season (Fortier et al. 2011). Having lower herbaceous biomass in the understory because of rapid canopy closure, the poplar buffer at Bromptonville might also have had lower C allocation to herbaceous plant roots, which may have contributed to the large C loss observed at this site, compared to the adjacent herbaceous buffer (Table 4). Root systems of understory plants play a major role in soil C cycling, in both young and older fast-growing plantations (Wu et al. 2011). The trend towards lower soil C in poplar riparian buffers may also be related to the export of a high amount of poplar leaf litter because of storm flow, flooding and wind, with few leaves reaching or remaining in the understory (J. Fortier, field observation). Because leaf litter also has a central role to play in forest soil development (Côté and Fyles 1994) and soil C storage in plantations (Laganière et al. 2010), its partial export outside the poplar buffers constitutes a net loss of an important input of organic matter to the soil.

On average, woodlots had more soil C in the 0–20 and 0–60 cm depth ranges than both types of buffers (Figure 5). This is because soil C stocks were particularly high in the 0–20 cm depth in the hemlock, sugar maple and cedar woodlots, but also in the 20–40 cm depth in the sugar maple woodlot (Figure 4). These tree species have an acidifying litter (Côté and Fyles 1994; Burns and Honkala 1990), which is consistent with the lower pH observed in the surface soil of the older woodlots, compared to riparian buffer soils (Table 1). Having lower pH near the soil surface, the older woodlots may have lower rates of organic matter mineralization (Paustian et al. 1997), and greater rates of soil C accumulation than agricultural buffers. This interpretation is supported by a significant negative relationship between soil pH and soil C concentration in the 0–20 cm depth range across all sites and all riparian land uses (Figure 8). Greater surface soil C concentrations and lower soil pH were also characteristic of natural woodlot soils when they were compared to adjacent abandoned fields and 10 year-old hybrid poplar plantations in southern Québec (Boothroyd-Roberts et al. 2013). The particularly high C stocks in the 0–20 cm layer of the hemlock woodlot is also consistent with the fact that eastern hemlock litter is highly refractory to decomposition (Elliott et al. 1993). In addition, even if the soil surface of older woodlots was much less compact than buffer soils (Table 1), these woodlots had greater soil C stocks in the 0–20 cm depth range (Figure 4) because soil C concentrations were particularly high in that surface soil depth range (43.8-75.5 g kg^-1^) (Table 3). Higher bulk density in buffer surface soil was probably the result of several years of livestock trampling and agricultural traffic (Willatt and Pullar 1984; Blackwell and Soane 1981) prior to buffer establishment. Greater soil C stocks and lower bulk density were also found in woodlot soils of the North Central United States compared to adjacent agricultural crops and poplar plantations (Coleman et al. 2004).

Finally, it should be mentioned that the particularly high variation in stone volume observed between poplar and herbaceous buffers at some sites (Magog and St-Isidore-de-Clifton) (Table 1) adds a great deal of variability to our soil C stock estimations. Small agricultural streams of southern Québec have often been straightened and dredged (Beaulieu 2001) and, as observed by the landowner of the St-Isidore-de-Clifton site, stones lying in the bottom of the stream have often been piled on stream banks (A. Doyon, pers. comm.). Soil stoniness at some sites also greatly complicates soil sampling. However, although costly and time consuming, stoniness estimations for stony soils is essential to increasing soil C estimate precision, because bulk density measurements with a soil corer alone will lead to overestimations (Andraski 1991; Vincent and Chadwick 1994; Throop et al. 2012).

Based on this study, the greatest benefits of hybrid poplar riparian agroforestry systems in terms of C storage is in the tree biomass, since soil C seems unaffected or depleted. With root biomass reaching 27.3 t ha^-1^ during the 9th growing season (Table 2), and aboveground woody biomass reaching 193 t ha^-1^ after 9 years (Fortier et al. 2013), hybrid poplar buffers clearly have the potential to increase C storage on farmland. Other C benefits of poplar agroforestry systems are the potential fossil fuel displacement by woody biomass production and the long-term storage of biomass C in solid wood products.

## Conclusion

This study suggests that the greatest benefits of establishing a hybrid poplar buffer in a riparian zone previously dominated by herbaceous vegetation are a large increase in coarse root biomass down in the soil profile, and an increase in fine root biomass at depth as well. Results also highlight the particular ability of poplar root systems to colonise deeper soil horizons when compared to native woodlot species. Conversely, lower or similar soil C stocks were found in poplar buffers in comparison to adjacent herbaceous buffers, especially near the soil surface, probably because poplars caused a reduction in fine root biomass in surface soil; an interpretation supported by a strong positive relationship between fine root biomass and soil C. Finally, on average, natural woodlot soils (never disturbed or undisturbed for several decades) tend to have greater soil C stocks than buffer soils, which were still agricultural soils less than 10 years ago.

## Material and methods

### Study sites and experimental design

This study took place in the southern region of the province of Québec, Canada. At the four study sites (Bromptonville, Magog, Roxton Falls and St-Isidore-de-Clifton) three types of riparian land uses were studied for root biomass and soil C stocks distribution: (1) hybrid poplar riparian buffer, (2) herbaceous riparian buffer and (3) natural riparian woodlot.

Three of the study sites (Bromptonville, Magog and Roxton Falls) are located in a hilly landscape (Sherbrooke unit), which is characterised by gentle slopes and a continental subhumid moderate climate (Robitaille and Saucier 1998). Land use in this landscape unit is 71% natural and managed forest (mostly private), 28% agriculture and 1% urban. Agricultural activities are concentrated in larger valley bottoms; pastures are frequently found on the poorer hillside soils. The St-Isidore-de-Clifton site is located in the Mont Mégantic landscape unit, which is characterised by continental subhumid-subpolar climate, higher elevation, steeper hillside slopes and lower agricultural land use (9% of land use) (Robitaille and Saucier 1998). Both landscape units are covered by a thick surface deposit of till and share a similar precipitation regime (1000–1100 mm). St-Isidore-de-Clifton, Magog, and Bromptonville sites are located in the St-François River watershed, while the Roxton Falls site is in the Yamaska River watershed. These watersheds both drain into the St. Lawrence River.

At each site, hybrid poplar riparian buffers where planted in spring 2003 at a density of 2222 stems per hectare on both sides of the streams for a total length of 90 m and a width of 4.5 m on each stream bank. Bare-root hybrid poplar plants were 1 year-old when they were planted. In the year of the study (2011), the buffers were in their 9th growing season. No site preparation was done prior to planting and tending operations consisted in a single localised herbicide treatment (1 m^2^/tree) in June 2003. Information regarding hybrid poplar buffer management, aboveground biomass and volume growth, aboveground nutrient and C accumulation, and understory biomass and diversity can be found in previous studies (Fortier et al. 2010a, 2010b, 2011; 2012;2013).

At each site, unmanaged (free-growing) herbaceous buffers were located within 100 m upstream or downstream of the hybrid poplar buffers. These herbaceous buffers generally consist of a mixture of native and exotic ruderal species that have naturally colonised the riparian zone, or that have been sown as pasture forage (Fortier et al. 2011). The dominant species (in percent coverage) in such buffers are *Phleum pratense, Agropyron repens*, *Agrotis* spp., *Vicia cracca*, and *Solidago* spp. The unmanaged herbaceous buffers were protected by a fence for at least two years at the three pasture sites to prevent livestock grazing.

At each site, a natural riparian woodlot, located as close as possible from both hybrid poplar and herbaceous buffers, was selected. These woodlots were located 1 km or less upstream of the poplar buffers. The 4 riparian woodlots were very different among the sites: (1) a 200 year-old eastern hemlock (*Tsuga canadensis*) dominated stand at Bromptonville; (2) a 73 year-old eastern white cedar (*Thuja occidentalis*) stand where livestock have complete access at Magog; (3) a 27 year-old grey birch (*Betula populifolia*) stand at Roxton Falls, and (4) a 54 year-old sugar maple (*Acer saccharum*) stand at St-Isidore-de-Clifton. The age of these stands was estimated by coring the dominant trees. Riparian land use characteristics are summarized in Table 5.

In the hybrid poplar buffer land use, a randomized block design was used at each of the 4 sites, with 4 blocks (replicates) and 3 hybrid poplar clones: (1) *P*. *deltoides* × *nigra* (DxN-3570; also named *P*. x *canadensis*); (2) *P*. *canadensis* × *maximowiczii* (DNxM-915508); and (3) *P*. *maximowiczi* × *balsamifera* (MxB-915311). A total of 48 hybrid poplar riparian buffer experimental plots were sampled in this study. These plots are 4.5 m wide and 9 m long (40.5 m^2^). Each plot contains 9 trees from a single clone (3 rows; 3 trees per row).

At each site, four herbaceous buffer plots were sampled (n = 16, 4 sites × 4 plots/site). The size of the herbaceous buffer plots was 4.5 m (poplar buffer width) × 9 m (40.5 m^2^). At each site, there were 4 riparian woodlot plots (n = 16, 4 sites × 4 plots/site). The size of these woodlot plots was 4.5 m × 9 m (40.5 m^2^).

In this study, the entire experimental design contains 80 experimental plots covering three types of riparian land uses: (1) 48 hybrid poplar riparian buffer plots; (2) 16 unmanaged herbaceous riparian buffer plots; (3) 16 natural riparian woodlot plots.

### Coarse and fine root sampling

Root sampling was done from mid-June to mid-July 2011. In each plot (n = 80), coarse root biomass (diameter > 2 mm) samples were obtained by excavating pits (50 × 50 cm by 60 cm deep) and harvesting all coarse roots in the pits. During the excavations, coarse root distribution was also measured for three soil depth ranges: (1) 0–20 cm, (2) 20–40 cm and (3) 40–60 cm. Coarse root samples where washed with water and air dried. Coarse root subsamples were collected to determine dry weight. In the hybrid poplar buffer and woodlot plots, the pits were located 25 cm away from a representative tree, so that coarse root samples did not include stump biomass. The representative tree was the closest to the average diameter at breast height (DBH) of all trees in the plot. Diameter at breast height ranges of the sampled trees for the hybrid poplar buffer and the woodlots at each site are given in Table 5. In the particular case of herbaceous buffers, roots having a diameter greater than 2 mm were herbaceous plant rhizomes and they will be considered as coarse roots in this study.

In each plot (n = 80), fine root biomass (diameter < 2 mm) samples were obtained by extracting two soil cores (core diameter = 5.3 cm, core length = 10 cm, volume of both cores = 220.6 cm^3^) from pit walls for each of three depth ranges (10–20, 20–40 and 40–60 cm). In each plot, two additional soil cores were randomly extracted vertically from the soil surface (0–10 cm), and combined with the two cores extracted from pit walls between 10–20 cm depth, in order to obtain a single fine root sample for the 0–20 cm depth range. For the 20–40 and 40–60 depth ranges, the two soil cores were combined to produce a single fine root sample per depth. Fine root biomass samples, which contained both live and dead fine root biomass, were separated from soil by hand picking, washed and dried at 65°C to determine dry weight.

### Mineral soil characteristics and carbon stocks and distribution

Soil sampling was done from mid-June to mid-July 2011. In each plot (n = 80), soil characteristics and carbon stocks were obtained by extracting two soil cores (core diameter = 5.3 cm, core length = 10 cm, volume of both cores = 220.6 cm^3^) from pit walls for each of three depth ranges (0–20, 20–40 and 40–60 cm). For each depth range, the two cores were combined to produce a single soil sample. In woodlot plots, the sampling protocol was slightly modified for the 0–20 cm layer in order to properly sample the A horizon, which was relatively thin at some sites. In woodlot plots, one core was extracted vertically from the soil surface (0–10 cm) and combined with another core extracted from pit walls between 10–20 cm depth. Soil samples were air dried and sieved (2 mm). Soil C concentrations were determined by the combustion method at high temperature (960°C) followed by thermal conductivity detection. These analyses were done by the CEF lab (Dr. R. Bradley and Dr. W. Parsons) at the University of Sherbrooke. Soil pH and texture were determined by the Agridirect Inc. soil analysis lab in Longueuil (Québec). Methods used are those recommended by the Conseil des productions végétales du Québec (1988). The determination of soil pH was made using a 2:1 ratio of water to soil. For particle size analyses, the Bouyoucos (1962) method was used. However, due to high analysis costs, particle size analysis was done on composite soil samples. In the poplar buffers, soil samples where pooled at the block level at each site (4 samples were analysed per site). For the herbaceous buffers, one composite soil sample was made at each site by combining soil samples collected in each replicate. The same procedure was used in woodlot plots.

Soil bulk density was determined by drying sieved soil at 105°C and dividing the soil dry mass by the volume of soil cores, as recommended by Throop et al. (2012). Stoniness was assessed visually, by at least two persons, from the soil pit excavation. For each sampling depth range, stones (larger than the core diameter) that were removed by excavation were replaced in the pit to estimate pit volume (in %) that was occupied by stones. In each plot and for each depth, C stocks and nutrient stocks were calculated by multiplying soil C and nutrient concentrations with soil mass, with respect to soil bulk density and stoniness.

### Forest floor sampling

In each woodlot plot that had a LFH Horizon (O Horizon), three LFH samples (50 × 50 cm) were collected at the end of July 2011. These LFH samples consisted essentially of dead tree leaves, and excluded fine and coarse woody debris. Subsamples were collected to determine dry weight and C concentrations and contents of the LFH layer.

### Statistical analyses

For data analysis related to hybrid poplars, ANOVA tables were constructed in accordance with Petersen (1985), and degrees of freedom, sum of squares, mean squares and *F*-values were computed. When a factor was declared statistically significant (Site, Clone and Site × Clone interaction), the standard error of the mean (SE) was used to evaluate differences between means for three levels of significance (*p* < 0.05, *p* < 0.01 and *p* < 0.001). All of the ANOVAs were run with the complete set of data (4 sites, 3 clones, 4 blocks = 48 experimental plots).

Given that no Clone effect and no Site × Clone interaction were detected by the ANOVA on root biomass and soil variables in the hybrid poplar experimental design, we have averaged root and soil variables of the 3 clones within a block, in order to produce data at the block level. Consequently, for statistical analysis, the number of plots in the hybrid poplar buffer land use type was reduced from 48 to 16 plots, which is equivalent to the number of plots found in the two other riparian land uses (herbaceous buffer and woodlot). Thereafter, a series of ANOVAs was used to evaluate the riparian Land use and Site effects and Land use × Site interaction on root biomass and soil C variables. The model for each ANOVA included 3 Land uses (hybrid poplar buffer, herbaceous buffer and woodlot) and 4 sites (Bromptonville, Magog, Roxton Falls and St-Isidore-de-Clifton) and 4 replicates of each riparian land use at each site (3 Land uses × 4 Sites × 4 replicates = 48 plots).

For the presentation of results in figures, abbreviations of the names of plantation sites were used (Bromptonville = Bro, Magog = Mag, Roxton Falls = Rox, St-Isidore-de-Clifton = Sti). Root biomass and soil carbon stocks data were scaled up to the hectare for comparison purposes with other studies.
